# Supplementary material for: Melatonin-Assisted Cisplatin Suppresses Urinary Bladder Cancer Cell Proliferation and Growth through Inhibiting PrPC-Regulated Cell Stress and Cell Proliferation Signaling
Source: Int J Mol Sci. 2023 Feb 8;24(4):3353. doi: 10.3390/ijms24043353 (PMC9959909; doi:10.3390/ijms24043353)

Supplementary Figure S1. Preliminarily tested the role of PrP^C on regulating the PI3K/Akt/m-TOR pathway-mediated MMP9

To elucidate the role of PrP^C on regulating the PI3K/Akt/m-TOR-MMP9 signaling, the Western blot analysis was performed (n=1). The preliminary result revealed that as compared with the control group (Clt), the protein expressions of PI3K, p-Akt, p-m-TOR and MMP9 were remarkably enhanced in S1 and more remarkably increased in S2, whereas these parameters were substantially reduced in S3 and S4, suggesting that PrP^C played an essential role on upregulating this cell proliferation signaling. PrP^C = cellular prion protein; MMP9 =matrix metalloproteinase 9.

Clt = SV-HUC-1; S1 = T24 cells only; S2 = PrP^C overexpression in T24 cells (denoted as $PrP^{C\text{-}OE}$-T24 cells); S3 = T24 cells with PrP^C silencing (denoted as siRNA-PrP^C in T24 cells); S4 [$PrP^{C\text{-}OE}$-T24 cells + LY294002 (20μM)]; note LY294002 is a broad-spectrum of PI3K inhibitor.

Supplementary Figure S2. Preliminarily tested the impact of melatonin and cisplatin on regulating the cell proliferation signaling in cancer cells

The Western blot analysis (n=1) demonstrated that the protein expressions of PI3K, p-Akt, p-m-TOR and MMP-9 were notably suppressed in T2 and more notably suppressed in T3, whereas the protein expression of P-TEN exhibited an inverse manner of cell proliferation signaling among the group.

T1 = T24 cells only; T2 = T24 cells + melatonin (100 uM) for 24h co-culture; T3 = T24 cells + cisplatin (6 uM) for 24h co-culture.

Supplementary Figure S1

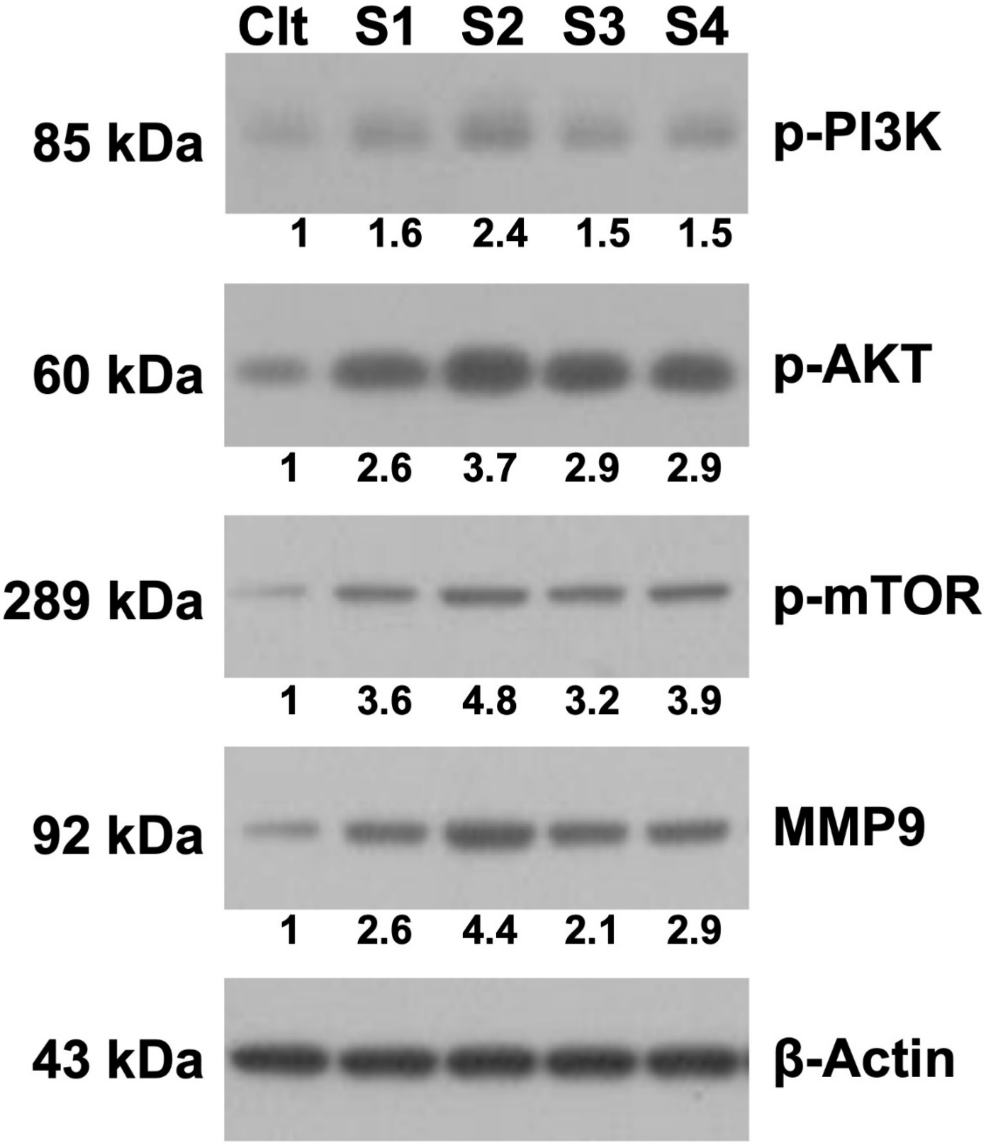

Supplementary Figure S2

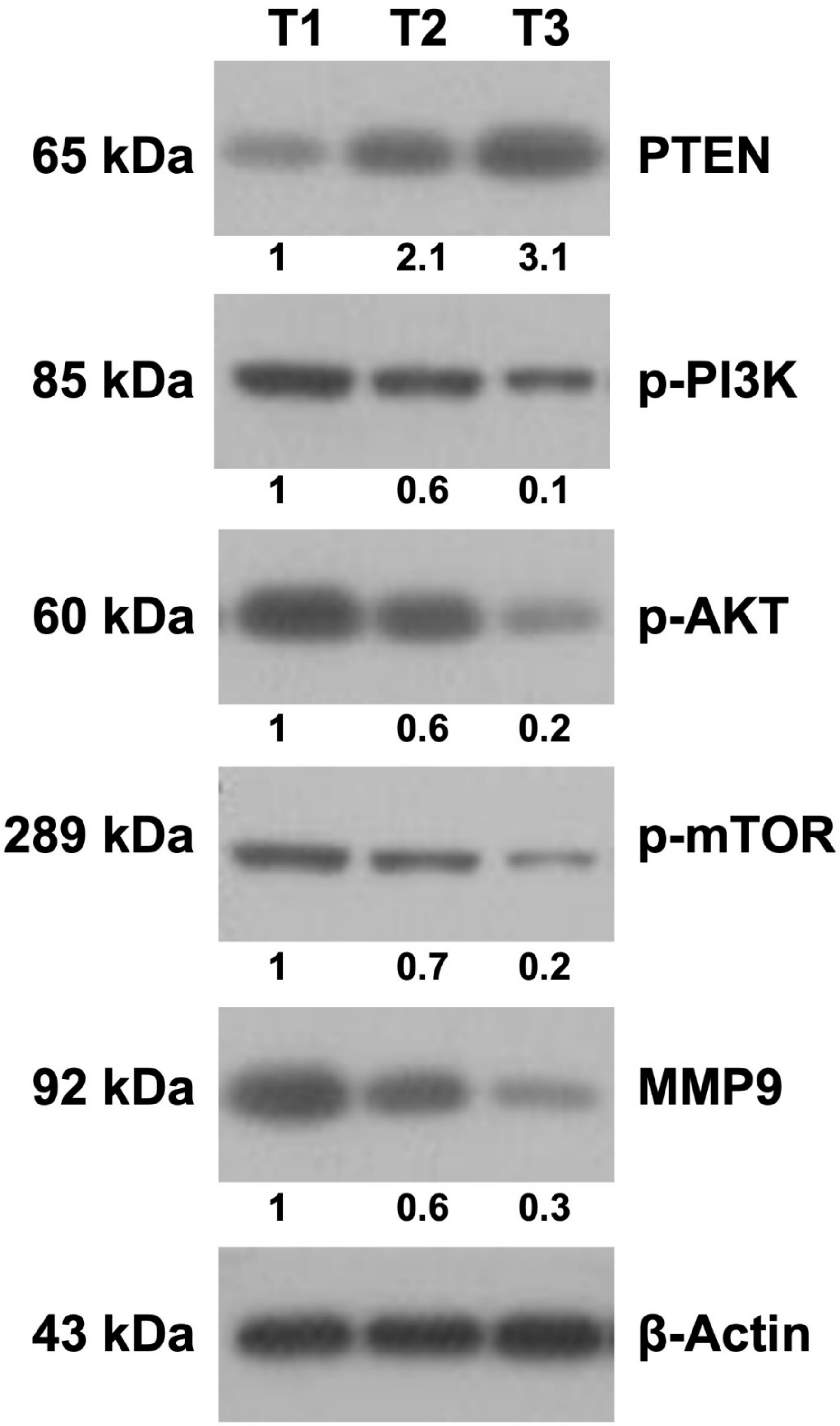

Supplement: Supplementary file 1 [file ijms-24-03353-s001.zip › ijms-2104245-supplementary.pdf]
